# The Bionanoprobe: hard X-ray fluorescence nanoprobe with cryogenic capabilities

**DOI:** 10.1107/S1600577513029676

**Published:** 2013-12-12

**Authors:** S. Chen, J. Deng, Y. Yuan, C. Flachenecker, R. Mak, B. Hornberger, Q. Jin, D. Shu, B. Lai, J. Maser, C. Roehrig, T. Paunesku, S. C. Gleber, D. J. Vine, L. Finney, J. VonOsinski, M. Bolbat, I. Spink, Z. Chen, J. Steele, D. Trapp, J. Irwin, M. Feser, E. Snyder, K. Brister, C. Jacobsen, G. Woloschak, S. Vogt

**Affiliations:** a X-ray Science Division, Advanced Photon Source, Argonne National Laboratory, Argonne, IL 60439, USA; b Applied Physics, Northwestern University, Evanston, IL 60208, USA; c Department of Radiation Oncology, Northwestern University, Chicago, IL 60611, USA; d Xradia Inc., Pleasanton, CA 94588, USA; e Department of Physics and Astronomy, Northwestern University, Evanston, IL 60208, USA; f Northwestern Synchrotron Research Center, Argonne, IL 60439, USA; g Chemistry of Life Processes Institute, Northwestern University, Evanston, IL 60208, USA

**Keywords:** Bionanoprobe, hard X-ray fluorescence microscopy, cryogenic capabilities

## Abstract

Hard X-ray fluorescence microscopy is one of the most sensitive techniques for performing trace elemental analysis of biological samples such as whole cells and tissues. Conventional sample preparation methods usually involve dehydration, which removes cellular water and may consequently cause structural collapse, or invasive processes such as embedding. Radiation-induced artifacts may also become an issue, particularly as the spatial resolution increases beyond the sub-micrometer scale. To allow imaging under hydrated conditions, close to the ‘natural state’, as well as to reduce structural radiation damage, the Bionanoprobe (BNP) has been developed, a hard X-ray fluorescence nanoprobe with cryogenic sample environment and cryo transfer capabilities, dedicated to studying trace elements in frozen-hydrated biological systems. The BNP is installed at an undulator beamline at sector 21 of the Advanced Photon Source. It provides a spatial resolution of 30 nm for two-dimensional fluorescence imaging. In this first demonstration the instrument design and motion control principles are described, the instrument performance is quantified, and the first results obtained with the BNP on frozen-hydrated whole cells are reported.

## Abstract 2

The Bionanoprobe has been developed to study trace elements in frozen-hydrated biological systems with sub-100 nm spatial resolution. Here its performance is demonstrated and first results reported.

## 1. Introduction

Synchrotron-based X-ray fluorescence microscopy (XFM) has opened remarkable opportunities for quantitative study of trace elements, particularly metals, within whole cells and tissue sections (Wagner *et al.*, 2005 ▶; Paunesku *et al.*, 2006 ▶; Fahrni, 2007 ▶; Finney *et al.*, 2007 ▶; Szczerbowska-Boruchowska, 2007 ▶; Ortega *et al.*, 2009 ▶; Paunesku *et al.*, 2009 ▶; Ralle & Lutsenko, 2009 ▶; de Jonge & Vogt, 2010 ▶; Fittschen & Falkenberg, 2011 ▶; Bohic *et al.*, 2012 ▶; Jensen *et al.*, 2012 ▶; Vogt & Ralle, 2012 ▶). By using spatially coherent highly brilliant X-rays from a synchrotron and advanced X-ray optics, current XFM instruments at the Advanced Photon Source (APS) at Argonne National Laboratory routinely achieve sub-micrometer probe size and are capable of detecting trace elements with a sensitivity down to parts per million (p.p.m.) in whole cells or tissue sections. In addition, chemical state information, such as the oxidation state of elements, can be elucidated using X-ray fluorescence spectroscopy and X-ray absorption near-edge structure (XANES) (Norman, 1986 ▶; Dyar *et al.*, 1998 ▶; Ortega *et al.*, 2009 ▶; Gorman-Lewis *et al.*, 2011 ▶; Bohic *et al.*, 2012 ▶; Jensen *et al.*, 2012 ▶; Vogt & Ralle, 2012 ▶; Oakes *et al.*, 2012 ▶).

XFM with a beam spot size below 50 nm has been pursued by several institutions with the aim of revealing and analyzing nanoscale structures and features in complex systems such as cells and tissues, advanced materials and devices (Bleuet *et al.*, 2009 ▶; Matsuyama *et al.*, 2010 ▶; Chu, 2010 ▶; Schroer *et al.*, 2010 ▶; Somogyi *et al.*, 2011 ▶; Winarski *et al.*, 2012 ▶; Maser *et al.*, 2013 ▶). However, conventional sample preparation methods are not sufficient, in many cases, to preserve the ultrastructure of biological samples in X-ray imaging (Schneider, 1998 ▶; Howells *et al.*, 2009 ▶). Room-temperature wet specimens are damaged by immediate radiation due to primary bond breaking in organic materials as well as hydrolysis of water, so that they suffer from shrinkage as well as material diffusion across compromised membranes. Dehydration by methods such as air-drying, freeze-drying and critical-point drying conveys increased robustness against radiation damage, but the loss of diffusible ions is possible during the dehydration process and dehydration can also cause cellular ultrastructure to shrink, distort or even collapse (O’Toole *et al.*, 1993 ▶; Williams *et al.*, 1993 ▶; Beveridge, 2005 ▶). A significant step towards accurate imaging of subcellular structures and elemental distributions is to rapidly cool fully hydrated samples ideally to a vitrified state, and image these samples under frozen-hydrated conditions. Such frozen-hydrated samples are considered more faithful to their natural states than chemically fixed samples, not only because cellular components such as organelles are fixed in their natural positions but because cellular water and ionic distributions are also retained (Vanhecke *et al.*, 2010 ▶).

Structural damage caused by ionizing radiation is another concern as spatial resolution increases. Dehydrated cells and tissues are more radiation resistant than wet samples by eliminating secondary chemical damage caused by free radicals in solution. However, high-resolution X-ray microscopy inevitably involves a high radiation dose, causing radiation-induced bond breaking and consequent mass loss (Kirz *et al.*, 1995 ▶; Beetz & Jacobsen, 2003 ▶; Nishino *et al.*, 2009 ▶). The effects of energy deposition can be minimized by preserving cells in their frozen-hydrated state and performing X-ray examinations close to liquid-nitrogen temperature (Sayre & Chapman, 1995 ▶; Maser *et al.*, 2000 ▶).

**Figure 1:**
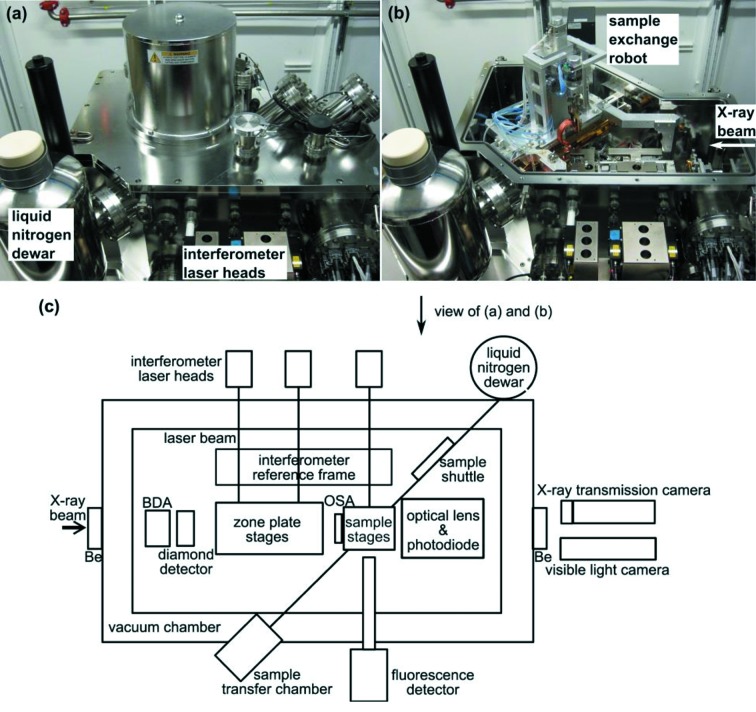
Photographs (*a*, *b*) and schematic (*c*) of the BNP. The BNP is a sample-scanning hard X-ray fluorescence nanoprobe with laser interferometer systems for accurate positioning and cryogenic capabilities dedicated to examination of frozen-hydrated biological samples. The monochromatic X-rays travel in and out of the vacuum chamber through beryllium windows. Fresnel zone plates are used as nanofocusing optics. While the sample is being scanned through the focused beam, fluorescence signals are collected using the detector mounted at 90° with regards to the incident X-ray beam. Downstream of the sample a quadrant photodiode is used to collect transmission signals for differential phase and absorption contrast imaging. The photodiode can also be moved out of the X-ray path to allow the use of either an X-ray transmission camera or a visible-light microscope outside the chamber for alignment. Liquid nitrogen is the primary cooling source. The sample stage, shuttle and robot gripper are conductively cooled by liquid nitrogen during operation.

We describe here an instrument, the Bionanoprobe (BNP) (Fig. 1 ▶), which is the first commercial instrument with cryo transfer and scanning X-ray fluorescence imaging capabilities. The instrument was developed by Xradia with important input from others on our team. The BNP is designed to transfer samples under cryogenic conditions in a preparation station onto a cartridge, with cryo transfer of this cartridge onto a cryogenic scanning stage for X-ray fluorescence experiments. Fresnel zone plates (ZPs) are used as nanofocusing optics; eight different optics are mounted simultaneously to allow fast switching between different spatial resolutions as well as incident X-ray energy settings, providing a spatial resolution down to 30 nm. In this manuscript we demonstrate the basic capabilities of the BNP and report our first observations on frozen-hydrated whole cells.

## 2. System design and set-up

**Figure 2:**
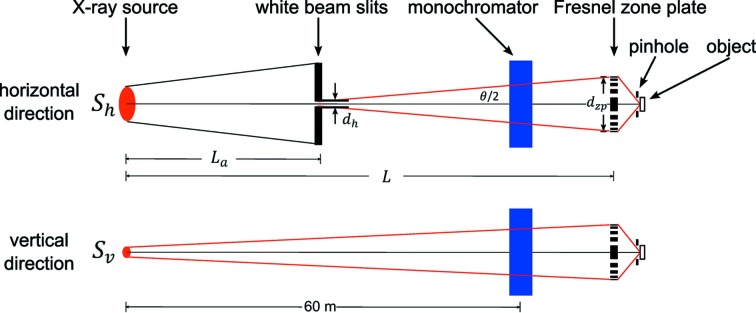
Schematic showing the optical layout of the beamline and the BNP (not to scale). *S*_h_ and *S*_v_ are the horizontal and vertical source sizes, respectively, while *d*_zp_ is the zone plate diameter. *L*_a_ is the distance from the source to the horizontal white-beam slits (with a width *d*_h_, which serve as a secondary X-ray source in the horizontal direction). *L* is the distance from the source to the zone plate. Adequate spatial coherence for nanofocusing is provided when the full source size multiplied by the full acceptance angle phase space area is no larger than the X-ray wavelength λ, or *d*_h_ *d*_zp_/(*L* − *L*_a_) < λ in the horizontal and *S*_v_ *d*_zp_/*L* < λ in the vertical direction. This condition is met by the APS source size in the vertical direction and can be reached in the horizontal direction by adjusting the width of the white-beam slits *d*_h_.

The BNP is designed for operation at an undulator beamline at the APS at Argonne National Laboratory. It is installed as one of several instruments in the D station at the Life Sciences Collaboration Access Team (LS-CAT), where it currently operates with 25–50% of the available beam time. The beamline, as its specifications listed in Table 1 ▶, provides hard X-rays with photon energies (*E*) in the range of 4.5–35 keV with an energy resolution (Δ*E*/*E*) of 2 × 10^−4^. This energy range allows mapping of most elements in the periodic table *via* *K*- and *L*-edge excitation and X-ray fluorescence detection. Fig. 2 ▶ shows the layout of the optics of the BNP and the LS-CAT beamline 21-ID-D. White-beam slits in both horizontal and vertical directions are installed at 27.2 m and 27.7 m from the undulator source, respectively, with the horizontal slits serving as a secondary X-ray source in the horizontal direction. To quantify spatial coherence, we use the phase space parameter, *p* = *d*θ/λ, as defined by Winn *et al.* (2000 ▶), where *d* is the source size, θ is the full-angle accepted, and λ is the wavelength of radiation. For *p* = 0.5, or *d*θ = λ/2, close to diffraction-limited resolution is obtained. For *p* = 1, the spatial resolution is reduced by approximately 20%, at a gain of 2× in focused flux. We use *p* = 1 as a requirement for our spatial coherence in the horizontal direction. This corresponds to a horizontal slit size of 30 µm for partially coherent illumination of a ZP with a diameter of 160 µm, located ∼65 m from the undulator source. To further increase the focused flux, the horizontal slits are frequently set to 80–100 µm, yielding *p* = 2.7–3.3. The vertical slits are typically left open (3 mm), as the vertical primary source size (25 µm FWHM at the undulator) is small enough to provide spatially coherent illumination with *p* = 0.5 to the ZP. A double-crystal Si 〈111〉 monochromator located at 60 m from the source is used to select the photon energy. Monochromatic beam then propagates into the BNP instrument located at 65 m from the source.

As shown in Fig. 1(*c*) ▶, most of the components of the BNP are enclosed in a vacuum chamber at a pressure of typically 10^−7^–10^−8^ torr. This eliminates convective heating of the cold samples, and reduces absorption of low-*Z* elemental fluorescence. X-rays enter and exit the chamber through beryllium windows. The instrument chamber houses an optics train and a sample stage assembly. These are mounted on a base plate, which provides stiff connections between optics and the sample, and which in turn is kinematically mounted on the bottom of the instrument chamber. This approach minimizes positional changes due to the change in pressure and resulting changes of the instrument chamber (Shu *et al.*, 2004 ▶, 2007 ▶). The optics train consists of, from upstream to downstream, beam-defining apertures, a diamond detector, ZPs, and order-sorting apertures with diameters of 35 µm and 50 µm. Eight ZPs have been mounted to focus the incident X-rays to focal spots of different sizes and optimize flux intensity at different beam energies. The sample stage assembly consists of stepper stages for travelling 4 mm in three orthogonal translations, two piezo-driven flexure stages with a travel range of 100 µm and 20 µm in the horizontal and vertical directions, respectively, and a rotation stage with 180° rotation range around a vertical axis. It is capable of automatically acquiring tomographic datasets. Position encoding is provided by a six-axis laser interferometer system (RLE20, Renishaw, UK), which measures the positions of both the sample stages (*X* and *Y*) and the ZP stages (*X* and *Y* at both the upstream and downstream ends of the stages) relative to an in-vacuum invar reference frame with accuracy of a few nanometers. According to interferometer-based position coordinates, the control system compensates the relative drift between the sample and the ZP in both horizontal and vertical directions. The difference in the measurements at the upstream and downstream ends of the ZP stage will be used to correct angular motions during stage translation. The sample is positioned into the focal spot and raster-scanned using either step-scan mode or fly-scan mode (continuous motion for the inner scan loop) for image acquisition. The use of fly scans allows surveying of a fairly large region on the sample with very large pixel count (typically ≤ 2000 × 2000 pixels) and low scan overhead (typically ∼2.5 s per row). Full X-ray fluorescence spectra are collected during both step and fly scans using a collimated four-element silicon drift detector (Vortex-ME4, Hitachi High-Technologies Science America, USA) with a total active area of 170 mm^2^ and maximum solid angle acceptance of ∼0.65 sr, and using xMAP readout electronics (XIA LLC, USA). The detector is mounted at 90° with regards to the incident X-ray beam to minimize collection of scattered X-rays (the incident X-rays are linearly polarized, so in-plane elastic scattering is minimized at 90°). The detection sensitivity increases with the atom number of the element. For 1 s acquisition, the minimum detection limits for calcium, iron and copper are approximately 4.0, 1.4 and 0.5 attograms, respectively, estimated by scanning a thin-film X-ray fluorescence standard (RF8-200-S2453, AXO DRESDEN GmbH, Germany) with a 10 keV monochromatic focused beam of 200 nm × 100 nm spot size. The lightest element detectable in typical samples is aluminium due to the absorption of fluorescence by the beryllium window on the detector. Downstream of the optics train and sample stage assembly, a quadrant photodiode (AXUV, Opto Diode, USA) allows differential phase contrast signals to be collected simultaneously with the X-ray fluorescence signal (de Jonge *et al.*, 2008 ▶; Hornberger *et al.*, 2008 ▶).

**Figure 3:**
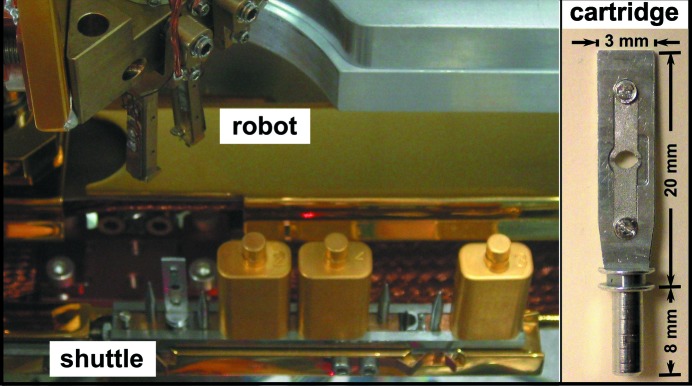
The sample exchange robot, shuttle and sample cartridges are shown on the left, while an example sample cartridge is shown on the right. Frozen biological samples are transferred under cryogenic conditions from an offline workstation into the BNP vacuum chamber. The robotic sample-exchange mechanism is designed to change samples in the BNP chamber while maintaining all the samples at a temperature below 110 K.

Cryogenic environment control and sample handling under cryogenic conditions are essential for studying frozen-hydrated samples. To avoid recrystallization of vitrified water, samples must be maintained at a temperature well below the glass transition of water at 135 K (for pure water) (Echlin, 1992 ▶) at all times. This requires both a cold stage on the BNP instrument and a cryo sample transfer mechanism designed to maintain the low temperature during sample exchange. By using an offline workstation, which can be situated away from the beamline in a sample preparation laboratory, delicate frozen samples are mounted on robust sample cartridges (Fig. 3 ▶) under liquid nitrogen, placed on a shuttle, and loaded into a transfer chamber. The transfer chamber is then used to deliver the shuttle into the BNP chamber through an air-lock system. This sample transfer mechanism enables four samples on individual cartridges to be loaded into the BNP vacuum chamber at one time. The use of cartridges minimizes the direct manual handling of samples, which greatly reduces the risk of contamination or damage of delicate TEM grids or silicon nitride windows. Each of the cartridges has a gold-coated cover, which further protects samples not in current use from radiative heating and frosting. A sample-exchange robot (Fig. 3 ▶) is used to grip cartridges from the shuttle and deliver them to the sample stage, with position reproducibility of a few micrometers (see video in the supporting information^1^). The sample stage is also cooled conductively from a liquid-nitrogen reservoir. All the procedures for loading and unloading samples are carried out under liquid-nitrogen temperatures to ensure that frozen samples are well preserved.

## 3. Motion control

Control of the BNP involves integration of a vendor-supplied instrument control system into an existing computing environment at the APS, with the ability to acquire fast fly scans with no per-pixel communication overhead losses and minimum per-scan-line losses. In order to reach these goals and to take advantage of key features of the EPICS control software (*e.g.* open source, networked, scalable, flexibility) (Experimental Physics and Industrial Control System, http://www.aps.anl.gov/epics/), Xradia has implemented control of the BNP using three EPICS IOCs (input/output controllers), two in PCs using Windows and one in a VME bus chassis using VxWorks as the operating system. Motion control is achieved using a Delta Tau Turbo PMAC2 Ultralite VME system (Delta Tau Data Systems, USA) interfaced from EPICS with the help of software jointly developed by the APS and the Diamond Light Source (http://www.gmca.aps.anl.gov/TPMAC2/, http://controls.diamond.ac.uk/downloads/support/tpmac/). The system provides a proportional-integral-derivative (PID) motion control of the sample stage motors based on reading back the sample position from the laser interferometer. The PID loop runs at 2 kHz speed, which makes it possible to implement on-the-fly scanning with a millisecond-range resolution. The fluorescence detectors are read by an XIA xMAP digital signal processor which is connected *via* fiber optic cable to a PCI card in a Windows PC. Detector triggers are generated at constant spatial intervals by the laser interferometer system with nanometer resolution. The actual dwell time per pixel is recorded from a constant frequency clock signal, which allows normalization of the data to count rate. A typical fly scan is acquired in the following manner:

(i) For a scan line, a starting position, pixel step size, number of pixels, pixel dwell time and a scan mode are sent by a client program (with a graphical user interface) to scan software running in the VxWorks IOC and the detector software running in a Windows IOC. The scan modes include the use of piezo stages for fine resolution scans (minimum pixel size of 5 nm) over short scan ranges (less than 100 µm) and the use of stepper motor stages for coarse resolution scans over longer ranges (between 0.1 mm and ∼4 mm).

(ii) The VxWorks IOC sends this information to the Delta Tau controller. The Delta Tau controller then uses this information to generate a motion profile which sets the acceleration and deceleration of the sample stages to be outside of the active scan area. The fast sample stage is moved according to this profile, while a hardware trigger generated by read-back of the laser interferometer is sent by the Delta Tau controller to the xMAP and a VME pulse counter to signal the start of new collection periods for the fluorescence and transmission detectors. A Struck multichannel scaler is used to collect the scaler information (incident beam flux, transmitted signals, *etc*.) at each scan position; the hardware trigger signal provides a channel advance; and the data for a whole line are stored in on-board memory. The xMAP also acquires the data for each scan line *via* the EPICS area detector software module (Rivers, 2010*a* ▶,*b* ▶).

(iii) At the end of the scan line, the current data stream is closed; data are read out from the multichannel scaler *via* the EPICS savedata task; and the fast sample stage is sent back to its original position. The client then requests a motion along the slowly varying scan axis (*e.g.* to the next scan row), and sends off a request to start another data stream to acquire a new scan line as above. The recently acquired scan line is displayed on the client screen showing the phase contrast image acquired using the transmission detector while the next line is being acquired.

This approach allows the BNP to be easily integrated into the standard EPICS beamline control environment of the APS, while allowing for the acquisition of fly scans with continuous motion and per-pixel times in the millisecond range, with the limitation currently being set by the readout speed of the electronics for the fluorescence detector.

## 4. Instrument performance at room temperature

**Figure 4:**
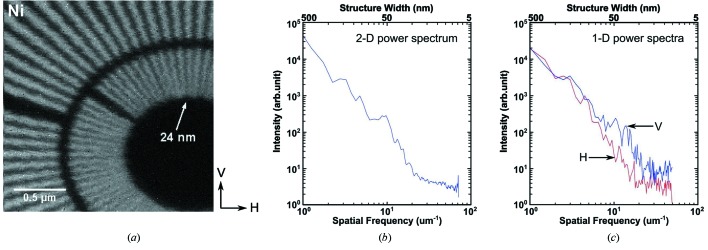
Images of a nickel (Ni) Siemens star test pattern to determine spatial resolution. (*a*) Ni fluorescence mapping of a 2 µm × 2 µm region acquired using fly-scan mode (continuous motion in the horizontal direction) with 10 nm pixel size and 30 ms dwell time per pixel. (*b*) Two-dimensional azimuthal power spectrum of the image indicating a cut-off spatial frequency of 20 µm^−1^, corresponding to a 25 nm half-period structure width. (*c*) One-dimensional power spectra of the image (horizontal direction in red, vertical direction in blue), indicating slightly better spatial resolution in the vertical direction.

To evaluate the spatial resolution of the BNP, a nickel (Ni) resolution test pattern with 24 nm inner spokes was imaged using monochromatic X-rays with a photon energy of 10 keV at room temperature. The test pattern was orientated at 0° rotation angle, *i.e.* perpendicular to the incident beam. A pair of stacked ZPs (Maser *et al.*, 2002 ▶; Snigireva *et al.*, 2007 ▶; Feng *et al.*, 2007 ▶; Chen *et al.*, 2008 ▶; Werner *et al.*, 2009 ▶) with 30 nm outermost zone width and 160 µm diameter (ZP30-160), with a combined thickness of 800 nm gold, were used to focus X-rays to a small spot. The diffraction efficiency for this pair of ZPs was measured to be 9.0% at a photon energy of 8.4 keV (Chen *et al.*, 2008 ▶). The horizontal white-beam slits were closed to 50 µm, yielding *p* = 1.7. The Rayleigh resolution of a fully coherently illuminated ZP30-160 is 36.6 nm. The value of the modulation transfer function would then fall to zero at a spatial frequency corresponding to a half-period of 15 nm, so that the cut-off frequency of ZP30-160 would be *f*_c_ = 33.3 µm^−1^ assuming incoherent image formation (such as in the case for a large-area transmission detector). For partially coherent illumination with *p* = 1, the cut-off would be at a slightly coarser spatial frequency. Fig. 4(*a*) ▶ shows the Ni fluorescence image of a 2 µm × 2 µm region of the central spokes on the test pattern obtained by performing a fly scan (continuous motion in the horizontal direction) with 10 nm step size and 30 ms dwell time for each step. Spatial frequency analysis was performed on the image. To avoid edge effects, a Gaussian edge-smoothing filter was applied before fast Fourier transform (FFT). As shown in Fig. 4(*b*) ▶, the two-dimensional azimuthal power spectrum indicates a cut-off frequency of 20 µm^−1^, corresponding to 25 nm half-period structure width, and consistent with partially coherent illumination. Spatial resolution could be further enhanced by closing down the white-beam slits and/or employing higher-resolution optics at the expense of usable flux. The spatial resolution was also studied separately for horizontal and vertical directions by computing one-dimensional power spectra (Fig. 4*c* ▶). Slight resolution degradation in the horizontal direction was observed, which can probably be attributed to the slightly lower coherence in the horizontal direction.

## 5. Thermal control and instrument performance in cryogenic conditions

**Figure 5:**
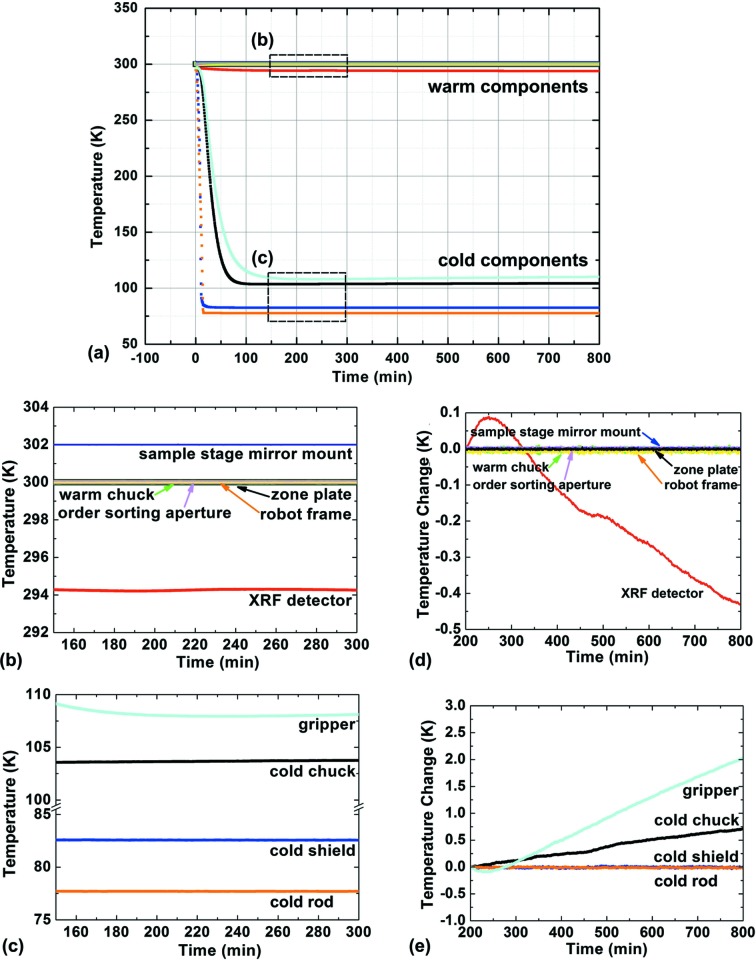
Graphs showing the temperatures of both cold and warm components recorded for ∼13 h starting with cooling of the BNP. The zoomed-in regions (*b*) and (*c*) show that both the warm and cold components achieved reasonably stable status after ∼2.5 h, with temperature changes between 200–800 min shown in (*d*) and (*e*). These measurements assure that the specimen can be kept below 110 K during cartridge changes, and for longer-term storage inside the vacuum chamber of the instrument. Temperature stability is also required to minimize specimen drift due to thermal expansion.

Thermal control for creating a stable cryogenic sample environment is critical for the BNP to achieve its performance target. While it is essential to sufficiently cool the components that are potentially in contact with samples, including the robot gripper, the cold sample chuck (the upper part of the sample stage that holds the sample), the cold shield surrounding the sample, and the sample shuttle, it is equally important to maintain neighboring components at room temperature to avoid radiative heat loss and thermal drifting. To this end, heaters with closed-loop control were implemented to regulate the temperature of various components that are essential to maintain thermal stability, including the sample stage mirror mount used for the interferometer system. To examine the thermal conditions, temperatures were recorded for ∼13 h starting when the liquid-nitrogen dewar was filled. As shown in Fig. 5 ▶, the warm components, except the X-ray fluorescence detector snout, were under closed-loop control and well regulated at ∼300 K with little variation [Figs. 5(*b*) and 5(*d*) ▶]. The temperature of the X-ray fluorescence detector snout, without being regulated, suggests that the ambient environment ∼5 mm away from the cold shield was a few degrees below 300 K and decreased by ∼0.5 K over the observation period. The cold components (conductively coupled to a liquid-nitrogen reservoir) rapidly dropped initially (Fig. 5*a* ▶) and became stable after ∼2.5 h, reaching a temperature below 110 K (Fig. 5*c* ▶). While the temperatures of the cold shield and cold rod (in direct contact with liquid nitrogen) show little variation over the observation period, the temperatures of the robot gripper and cold sample chuck (cooled by conduction through flexible braids) slightly increased. It is likely that the gripper might accumulate some heat from its holding current, and it is also possible that slight frosting of surfaces increases radiative heat transfer over time.

**Figure 6:**
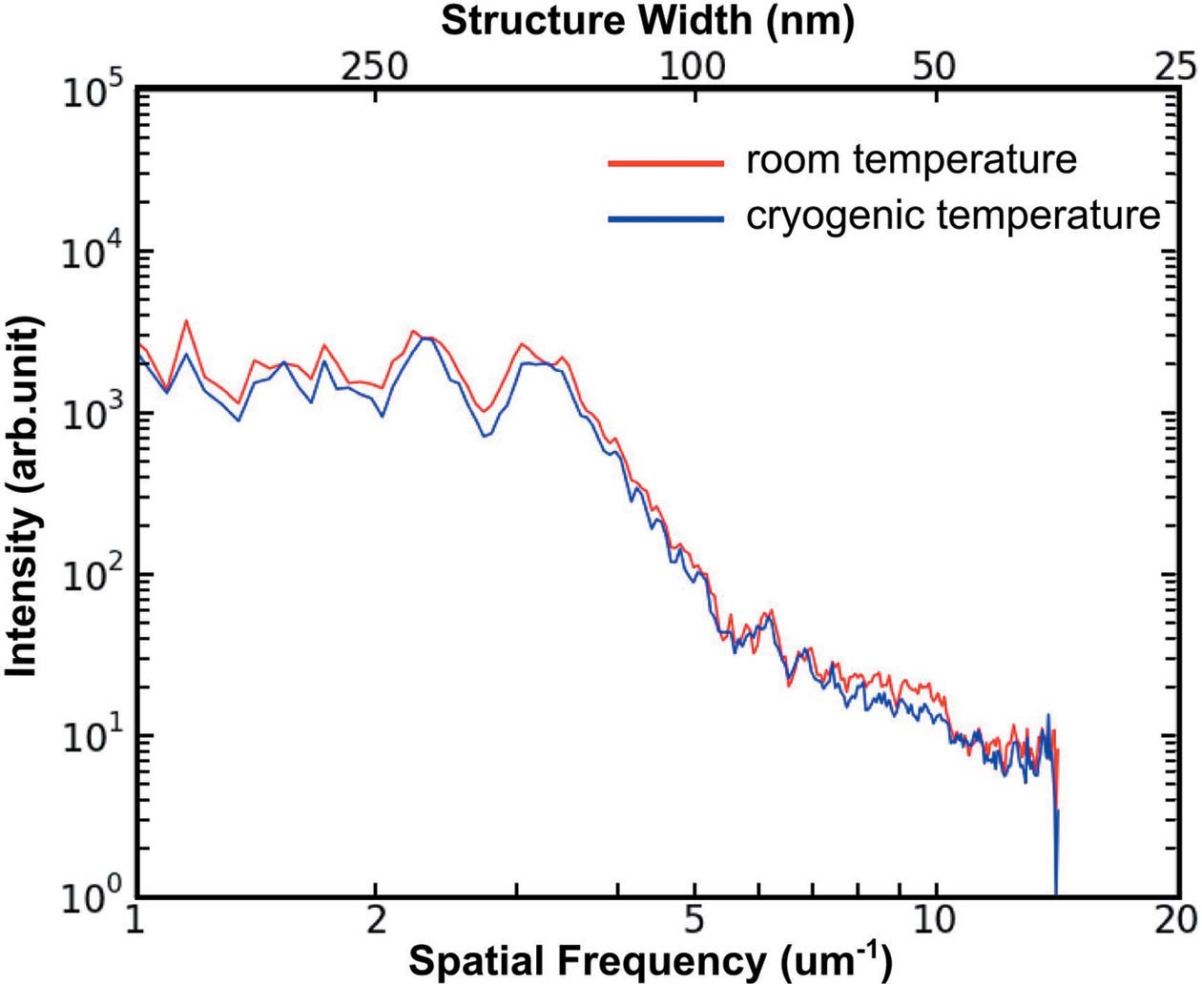
Two-dimensional azimuthal power spectra of two fluorescence images of the central spokes of a Ni test pattern that were obtained using identical scanning parameters (ZP70-160, 50 nm step size and 50 ms dwell time per pixel) at different thermal conditions (room temperature in red, cryogenic conditions in blue). This indicates that the BNP is able to deliver images with the same spatial resolution under both thermal conditions.

With the thermal conditions of the system carefully monitored and regulated, no measurable spatial resolution degradation was observed in scans performed using 50 nm step size at cryogenic *versus* room-temperature conditions. As shown in Fig. 6 ▶, two-dimensional azimuthal power spectra were computed from two images acquired using stacked ZPs with 70 nm outermost zone width and 160 µm diameter (ZP70-160), with 50 nm step size and 50 ms dwell time per pixel. The similar signals at high spatial frequencies indicate that the resolution of the two images is almost the same.

**Figure 7:**
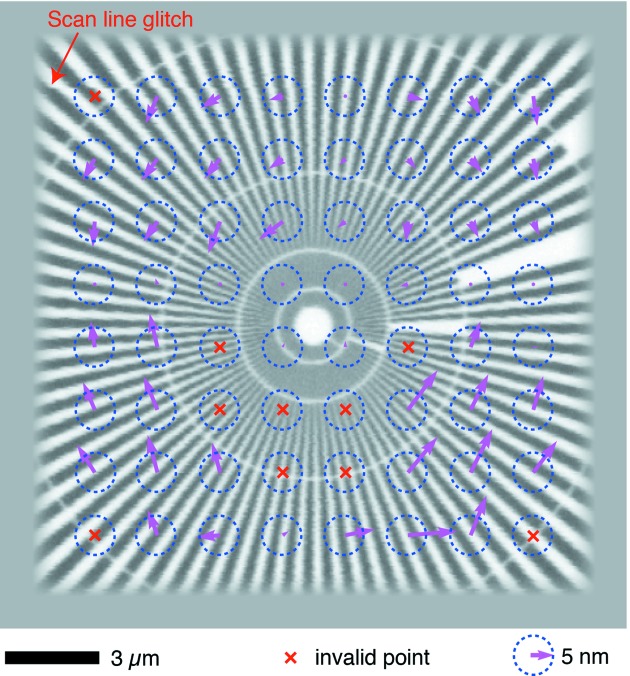
Image field distortion map of a test pattern imaged at cryogenic temperatures showing sub-15 nm field distortion. A Ni test pattern was imaged twice using the Ni fluorescence signal (both images had 50 nm step size and 50 ms per-pixel dwell time). The two images were then aligned by cross correlation from the center of the image, yielding an overall shift of the scan field of 2.8 pixels or 140 nm. After shifting the second image to correct for that overall shift, the image was broken up into 8 × 8 subfields. Sub-pixel-resolution cross correlation was used to measure the shift of subfields between the two images. The resulting magnitude and direction of the shift was plotted as an arrow with scaled length (scaled to 5 nm shift radius represented by the blue dashed circle). About 85% of these subfield shifts are less than 15 nm. This test shows that there is small relative distortion between images, allowing for consistent registration of images in applications such as spectromicroscopy and tomography.

Scanning repeatability is another factor of great importance. Any misalignment or distortion during a scan will result in image artifacts and consequently affect understanding of the samples. Such artifacts will also create unnecessary difficulties in reconstructing fluorescence tomographic datasets (currently under commissioning at the BNP). The repeatability was examined by subsequently performing two identical scans on the Ni test pattern using ZP70-160. The scans covered an 18 µm × 18 µm region and were acquired using fly-scan mode (continuous motion in the horizontal direction) with 50 nm step size and 50 ms dwell time per pixel. Each of these scans took ∼2 h. The differences can be classified as a result of overall image translation and image distortion. The effect of translation was quantified by cross-correlating these two images, which yielded a shift of 2.8 pixels or 140 nm. This overall shift, probably caused by slight temperature change of the cold chuck (Fig. 5*e* ▶) and consequent thermal expansion above the location of the laser interferometer mirrors, is easily compensated for by image registration. Distortion analysis was then performed on the registered images. The central areas of the two images were divided into 8 × 8 sub-regions. Sub-pixel registration was carried out with a zero-padded FFT approach on the corresponding sub-regions (Guizar-Sicairos *et al.*, 2008 ▶), resulting in 64 vectors indicating the local offsets. To visualize these offsets as a function of location, a so-called image field distortion map was generated (Fig. 7 ▶), where arrows were used to display vectors showing the local shifts. The magnitude of ∼85% of these vectors is less than 15 nm, indicating that the scanning motion is repeatable without significant distortion even under cryogenic temperatures. A scan line glitch, as labeled in the image, could also contribute to local shifts for distortion analysis. Such a glitch is very likely caused by inaccurate stage positioning in the vertical direction when compound motion took place (where the stepping motor stage is periodically moved one way while the piezo stage compensates, yielding a larger field of view than the piezo stage alone can provide), and can be prevented by fixing the stepper stage at the cost of the travel range.

## 6. Initial results of imaging frozen-hydrated whole cells

Sample preparation was carried out in a cryo-laboratory at the APS. Frozen-hydrated cells were obtained by plunge freezing in liquid ethane to lock diffusible ions in place and to minimize structural damage that would otherwise be caused by the formation of large ice crystals. The samples were then transferred within liquid nitrogen to the BNP workstation and loaded onto the sample cartridges. For X-ray examinations, the horizontal white-beam slits were closed to 80 µm to balance between the incident flux and spatial resolution. By using 10 keV monochromatic radiation and ZP70-160, the focused flux was measured to be ∼3.5 × 10^9^ photons s^−1^. Two applications are shown below.

(i) Green algae have been studied previously in a number of unique scientific contexts, such as biomineralization (Krejci *et al.*, 2011 ▶), their potential use for carbon sequestration (Twining *et al.*, 2003 ▶), and for production of biofuels (Rismani-Yazdi *et al.*, 2011 ▶). By using XFM with 400 nm spatial resolution, a recent study on dehydrated freshwater diatom revealed surprising metal distributions, particularly the ring-like structures formed by the distributions of manganese and iron in the siliceous cell wall (de Jonge *et al.*, 2010 ▶). To further understand the linkages of these metals and their functions on the molecular level in the organism, nanoscale mapping of elemental distribution within the cell preserved in its frozen-hydrated state is required. Here, frozen-hydrated *Chlamydomonas reinhardtii* (single-cell green algae) cells were examined using the BNP as a demonstration. We avoided structural and elemental alteration by immobilizing the whole cells through rapid freezing in liquid-nitrogen-cooled liquid ethane, without use of any chemical fixatives.

**Figure 8:**
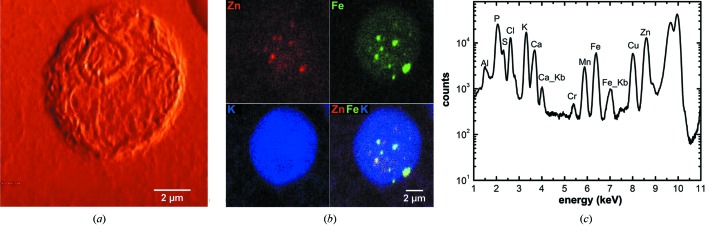
Images of a frozen-hydrated algae cell (*Chlamydomonas reinhardtii*) obtained using the BNP. (*a*) Differential phase contrast image showing some cell ultrastructure. (*b*) X-ray fluorescence images showing the distributions and the overlay of zinc (Zn), iron (Fe) and potassium (K). The count levels (minimum to maximum range in counts s^−1^) are 0–714 for Zn, 0–378 for Fe and 0–899 for K. The algae sample was plunge frozen in liquid ethane and stored in liquid nitrogen for several weeks before examination by the BNP. The images were acquired using fly-scan mode (continuous motion in the horizontal direction) with 35 nm step size and 250 ms dwell time per pixel. One of diffusible ions, K, shows a slightly uneven distribution in the cell demonstrating good cryogenic sample preparation and handling. The fluorescence maps were created by performing peak area fitting for every pixel. (*c*) The summed spectrum of the whole map with major peaks labeled. While the *K*α lines are labeled with element symbols only, the *K*β lines are indicated using ‘Kb’ in the graph.

The wild-type strain of *Chlamydomonas reinhardtii* Dangeard (ATCC No. 18798, USA) was grown mixotrophically in a Tris-Acetate-Phosphate (TAP) medium (Gorman & Levine, 1965 ▶) at 296 K under constant illumination at a rate of 100 µmol m^−2^ s^−1^ and with continuous shaking of 120 r.p.m. The algae cells were harvested by centrifugation at 500 g for 5 min when they reached a density of 2 × 10^6^ cells ml^−1^. Pelleted algae cells were resuspended in an equal volume of fresh TAP medium. 3 µl of the cell suspension was then deposited on a formvar-carbon-coated 200 mesh gold grid (EMS, USA). The grid was rapidly plunged into liquid ethane using an FEI Vitrobot Mark IV plunge freezer without any intermediate preparation step and then transferred into the BNP. By using this method, the sample can be cooled down to 90 K with a rate of 10^3^–10^4^ K s^−1^ (Maser *et al.*, 2000 ▶), preventing diffusion of small molecules and formation of ice crystals. Both differential phase contrast and fluorescence images of a single cell were acquired simultaneously by raster scanning the cell through the focused X-ray beam (10 keV monochromatic X-rays focused by ZP70-160) in 35 nm steps. Some ultrastructure was visible in the differential phase contrast image (Fig. 8*a* ▶). X-ray fluorescence images show that potassium (K) is almost evenly distributed throughout the cell, as would be expected, while zinc (Zn) and iron (Fe) have patterned distributions, co-localizing to some extent, probably in the context of the same subcellular compartments (Fig. 8*b* ▶). Data analysis, including both per-pixel fitting and elemental content quantification, was performed using *MAPS* software (Vogt, 2004 ▶), where the spectrum from each pixel collected from the sample was fitted with modified Gaussians (Van Grieken, 1992 ▶). For quantification, fluorescence measurements were compared with a calibration curve derived from measurements of a thin-film X-ray fluorescence standard (RF8-200-S2453, AXO DRESDEN GmbH, Germany).

(ii) TiO_2_–DNA nanocomposites (NCs) have been recently investigated as tools for nanobiotechnology (Paunesku *et al.*, 2003 ▶, 2007 ▶); ‘next generation’ nanoparticles made as composites of Fe_3_O_4_ and TiO_2_ (Fe_3_O_4_@TiO_2_ nanocomposites) have been developed as well (Arora *et al.*, 2012 ▶; Yuan *et al.*, 2013 ▶). Imaging of nanoparticle distribution in cells by XFM has been an essential aspect of this research (Paunesku *et al.*, 2003 ▶, 2007 ▶; Arora *et al.*, 2012 ▶; Yuan *et al.*, 2013 ▶). This study has significantly benefited from the use of the BNP because of its high spatial resolution. By using sub-100 nm beam size, both the nanocomposite distribution and subcellular compartments have been resolved. In this instance, HeLa cells were grown on 1.5 mm × 1.5 mm Si_3_N_4_ windows (Silson, UK) in Eagle’s minimum essential medium (EMEM) supplemented with 10% fetal bovine serum and 1% penicillin/streptomycin at 310 K and 5% CO_2_. After treatment with Fe_3_O_4_@TiO_2_ nanocomposites in serum-free media at 310 K to allow for nanocomposite internalization, HeLa cells were fixed with 4% formaldehyde as an approach for downgrading the sample to biosafety level 1 status. The sample was then plunge frozen to preserve the three-dimensional structure of the cells and allow investigation of the nanocomposite distribution and their spatial distribution in different subcellular compartments.

**Figure 9:**
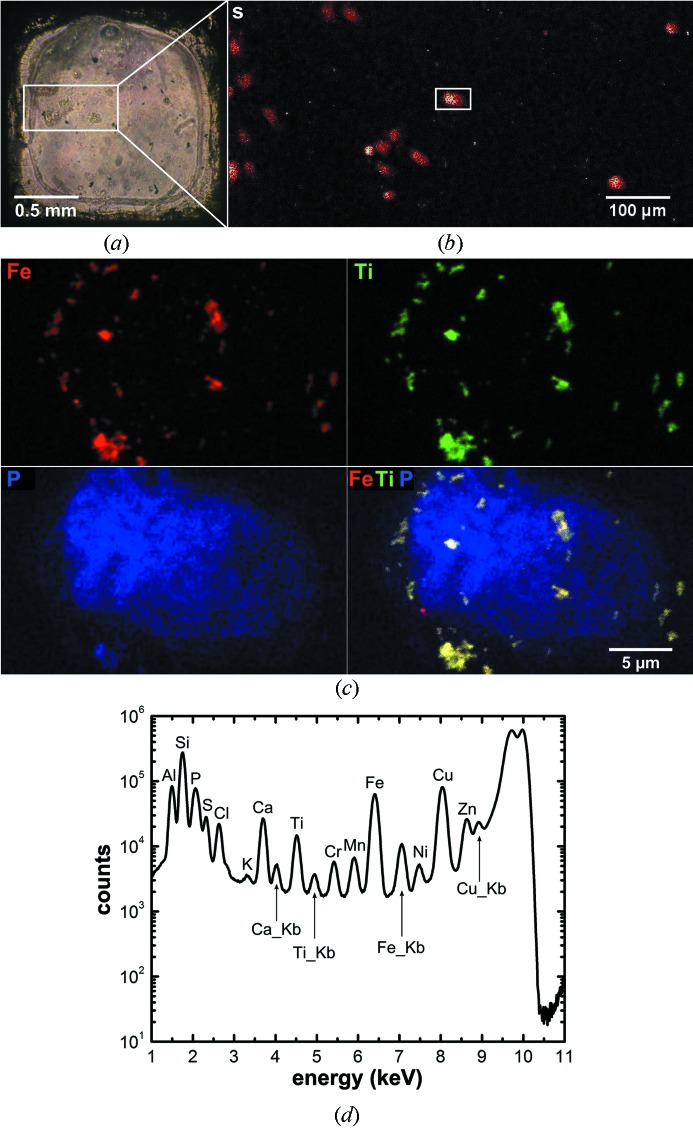
Images of a frozen-hydrated and chemically fixed HeLa cell sample that was treated with Fe_3_O_4_@TiO_2_ nanocomposites for 30 min. The nanocomposites consist of Fe_3_O_4_@TiO_2_ nanoparticles and peptides. The size of the Fe_3_O_4_@TiO_2_ nanoparticles is 6–7 nm, measured using atomic force microscopy. The peptides are 11 amino acids long with an N-terminal 3,4-dihydroxyphenyacetic acid moiety. (*a*) Overview optical image of the frozen-hydrated sample obtained using a Nikon light microscope equipped with an Instec cold stage. A 750 µm × 350 µm area of this sample, indicated by the white rectangle in (*a*), was rapidly scanned by the BNP to gain an overview of the distribution of cells in this sample. (*b*) Sulfur (S) fluorescence map of the sample area indicated in (*a*), acquired using fly-scan mode (continuous motion in the horizontal direction) with 0.85 µm step size. The count level (minimum to maximum range in counts s^−1^) is 0–100. The S signal indicates the presence of the cells. A 30 µm × 18 µm area, indicated by the white rectangle in (*b*), encompasses a single cell. (*c*) High-resolution fluorescence maps of the cell selected in (*b*) showing the distributions of iron (Fe), titanium (Ti), phosphorus (P) and their overlay. This image was acquired using fly-scan mode (continuous motion in the horizontal direction) with a 50 nm step size and 100 ms dwell time per pixel. The count levels (minimum to maximum range in counts s^−1^) are 0–705 for Fe, 0–569 for Ti and 0–124 for P. While P shows a cell outline, and a more intense P concentration in the region of the cell nucleus, co-localized Ti and Fe pixels correspond to the distribution of nanocomposites. The fluorescence maps were created by performing peak area fitting for every pixel. (*d*) The summed spectrum of the whole map with major peaks labeled. While the *K*α lines are labeled with element symbols only, the *K*β lines are indicated using ‘Kb’ in the graph.

Before transfer to the BNP, the frozen sample was examined using a Nikon 50i light microscope equipped with an Instec cold stage to locate regions of interest (Fig. 9*a* ▶). In the BNP, a 750 µm × 350 µm area was surveyed using 10 keV monochromatic radiation with 850 nm step size. Such large-area scan with sub-micrometer pixel size is only practically possible due to the use of fly-scan mode. Again, per-pixel fitting and elemental content quantification were performed using *MAPS* software. The fluorescence map of sulfur (S) was used to identify the cells (Fig. 9*b* ▶), since S is an integral part of cysteine and methionine residues present in proteins throughout the cell. By performing a finer resolution scan across one of the cells using ZP70-160 and 50 nm step size (Fig. 9*c* ▶), the distribution as well as the morphology of the nanocomposite aggregates, *i.e.* titanium (Ti) and iron (Fe) rich spots, was resolved. The co-localization between the phosphorus (P) rich region, most likely the nucleus, and Ti and Fe suggests the probable nuclear accumulation of the nanocomposites. Most importantly, the ongoing development of the BNP will allow further evaluation of the spatial relations between the nanocomposites and the different subcellular compartments by offering the capability of fluorescence tomography (Yuan *et al.*, 2013 ▶).

## 7. Summary

We have developed the Bionanoprobe, a scanning hard X-ray fluorescence nanoprobe with cryogenic capabilities, to facilitate mapping and quantification of trace elements in biological samples close to their natural state at nanometer-scale spatial resolution. The BNP is a significant advancement for studies that require the combination of thick frozen-hydrated samples (*e.g.* full cells), high lateral spatial resolution and trace element sensitivity. In this manuscript we have described the system design and reported the performance of the instrument at both room temperature and at cryogenic temperatures. We have demonstrated resolution down to 25 nm half-period structure width at room temperature and a good scanning repeatability under cryogenic scanning conditions during the first year of instrument commissioning. We have also shown the capabilities of the BNP for examining frozen-hydrated whole cells at sub-50 nm spatial resolution. The cryogenic capabilities of the BNP, along with its very high spatial resolution, hard X-ray capabilities, high-throughput and motion stability will enable us to address a wide range of challenges in life sciences and biological research.

## Supplementary Material

Video showing how the robot delivers a sample to the scanning stage in the Bionanoprobe vacuum chamber. DOI: 10.1107/S1600577513029676/pp5038sup1.wmv

## Figures and Tables

**Table 1:** Specifications of beamline 21-ID-D at the Life Sciences Collaboration Access Team

Source	U33S
Undulator period (mm)	33
Number of undulator periods	60
Source size at 8 keV (µm)	σ_*x*_: 277; σ_*y*_: 11
Source divergence at 8 keV (µrad)	σ_*x*′_: 11.7; σ_*y*′_: 3.9
Monochromator	Double-crystal Si 〈111〉
Energy range (keV)	4.5–35
Energy resolution (Δ*E*/*E*)	2 × 10^−4^

## Footnotes

1 Supporting information for this paper is available from the IUCr electronic archives (Reference: PP5038).
